# Modifications and optimization of manual methods for polymerase chain reaction and 16S rRNA gene sequencing quality community DNA extraction from goat rumen digesta

**DOI:** 10.14202/vetworld.2018.990-1000

**Published:** 2018-07-27

**Authors:** Durgadevi Aphale, Aarohi Kulkarni

**Affiliations:** 1 Praj Matrix, R & D Center, Division of Praj Industries Ltd., 402/403/1098, Urawade, Pirangut, Mulshi, Pune, Maharashtra, India; 2 Department of Health and Biomedical Sciences, Symbiosis International University, Gram Lavale, Mulshi, Pune, Maharashtra, India

**Keywords:** 16S rRNA gene sequencing, community DNA extraction, goat, polymerase chain reaction, rumen digesta

## Abstract

**Background and Aim:**

A critical prerequisite for studying rumen microbial community by high throughput molecular biology methods is good quality community DNA. Current methods of extraction use kits designed for samples from the different origin for rumen. This puts stress on the development of a relevant manual method for DNA extraction. The objective of this study was to modify the existing methods of community DNA extraction and thereby systematic comparison of their efficiency based on DNA yield, purity, 16S rRNA gene sequencing, and identification to determine the optimal DNA extraction methods whose DNA products reflect targeted bacterial communities special to rumen.

**Materials and Methods:**

Enzymatic method, Chemical method, Enzymatic + Chemical method, and Enzymatic + Chemical + Physical method were modified toward evaluation of community DNA extraction from solid, squeezed, and liquid fractions of goat rumen digesta. Each method was assessed critically for nucleic acid yield and its quality. The methods resulting in high nucleic acid yield, optimal purity ratios with intact band on agarose gel electrophoresis were optimized further. Optimized methods were studied using standard polymerase chain reaction (PCR) with universal bacterial primers and 16S rRNA primers of targeted rumen bacteria. Methods denoting the presence of targeted rumen bacteria were assessed further with 16S rRNA gene sequencing and identification studies. It led toward methods efficacy estimation for molecular biology applications. Effect of rumen sample preservation on community DNA extraction was also studied. Their mean standard deviation values were calculated to understand sampling criticality.

**Results:**

Modified Chemical method (Cetrimonium bromide) and Enzymatic+Chemical+Physical (ECP) method (Lysozyme-Cetrimonium bromide-Sodium Dodecyl Sulfate-freeze-thaw) could extract 835 ng/µl and 161 ng/µl community DNA from 1.5 g solid and 2 ml squeezed rumen digesta with purity ratios of 1.8 (A_260__nm_/A_280 nm_) and 2.3 (A_260__nm_/A_230 nm_) respectively. Comparative analysis showed the better efficiency of ECP method and chemical method toward freshly squeezed rumen digesta and solid rumen digesta. However, sample preservation at −80°C for 1.5 months drastically affected the yield and purity ratios of community DNA. New protocol revealed targeted microbial community having Gram-positive as well as Gram-negative bacteria such as *Prevotella ruminicola*, *Streptococcus lutetiensis*, *Ruminococcus flavefaciens, Fibrobacter succinogenes*, and *Selenomonas ruminantium*.

**Conclusion:**

To date, this is the first report of modified methods wherein least chemicals and steps lead toward PCR and 16S rRNA gene sequencing quality community DNA extraction from goat rumen digesta. Detection of targeted rumen bacteria in solid and squeezed rumen digesta proves their strongest association with rumen fiber mat. It also marks the presence of distinct microbial communities in solid and squeezed rumen fractions that in turn differs the performance of each different method employed and yield of nucleic acid obtained. It also leaves a possibility of the presence of complex microbial consortia in squeezed rumen digesta whose DNA extraction methods need more attention. Finally, manual protocols of community DNA extraction may vary in different ruminant which suggests undertaking rigorous research in their establishment.

## Introduction

The rumen microbial community is highly complex. There are approximately 10^11^ microbial cells per gram of rumen contents, and these belong to many different species and genera of bacteria, Archaea, fungi, ciliate protozoa, and viruses [1,2]. To date, relatively few of these have been successfully cultured and characterized. The classical approaches to understanding the rich microbial diversity are heavily dependent on their ability to grow on certain synthetic media. This, in itself, is a major limitation that provided the basis for the choice of molecular analyses of rumen microbial communities, especially the uncultivable microbes. Molecular analyses, in fact, allow us to detect the uncultured microbial community thus yielding insight into the vastly unknown regime of rumen flora. Such kind of analyses is crucial then to determine shifts that occur within the mega microbial communities of the rumen due to external influences such as feed and water. One of the techniques that make large-scale community analysis possible is high-throughput sequencing. It allows the study of extremely subtle effects of relative microbial community’s compositional changes to be precisely identified in terms of absolute and relative microbial marker loci [1]. For all such crucial studies, the common prerequisite of high quality and yield DNA stands true.

DNA quality and yields, even though a critical prerequisite are highly restricted since the extraction methods do not work equally efficiently for diverse microbial groups. To date, several studies have shown that the DNA extraction method used has an impact on the microbial community representation in samples from different habitats, including the rumen. The sampling technique used from a wide choice such as oral stomach tubing, sample collection through a rumen fistula, as well as fractionation of rumen sample (into, e.g., liquid and solid), can also have an impact on microbial community parameters [1,2]. To minimize the variation introduced by differing methodologies, Henderson *et al*. [3] used PCQI method for community DNA extraction and high-throughput sequencing. While the advanced new generation sequencing methods have brought a much deeper insight into the complexity of rumen system and substantially increased knowledge related to rumen microbial diversity [4] sufficient amount of high-quality DNA as an impediment needs to be catered to.

To enable a direct comparison of the rumen community structure from different individual samples, standardization of DNA extraction methods is crucial. Several DNA extraction methods, including commercial kits, have been tried for ruminal DNA extraction [5-7]. However, commercial kits for DNA extraction are designed for non-ruminal samples and thus inherently face a challenge of effectiveness and reliability for cross usages. Vaidya *et al*. [8] found very clear impact of DNA extraction methods, the selected rumen fractions on downstream analysis of rumen microbial community including relative abundances of specific community members. Tatiana *et al*. [9] demonstrated the impact of storage of rumen cud on yield of DNA for metagenomics, abundances of specific phyla, class, or other taxa which ultimately impact diversity indices and community richness. Here, we want to systematically compare the effectiveness of a variety of community DNA extraction methods for goat rumen digesta based on the DNA integrity, yield, purity and 16S rRNA gene sequencing results.

Present study evaluates the performance of modified Chemical method (CTAB), Enzymatic method (EM) (lysozyme-SDS-proteinase K), Enzymatic+ Chemical method (lysozyme-SDS-proteinase K+ CTAB), and Enzymatic+ Chemical+ Physical method (lysozyme-SDS-CTAB-Polyvinylpyrrolidone-freeze-thaw) for community DNA extraction from goat rumen digesta. Targeted bacteria such as *Ruminococcus flavefaciens* and *Streptococcus bovis* are Gram-positive, which make it difficult to extract their DNA effectively using the manual community DNA extraction protocols. Further, extracted DNA should carry good quality and its amenability for standard polymerase chain reaction (PCR) and 16S rRNA gene sequencing is most important prerequisite.

The current research was aimed to modify the existing methods of community DNA extraction and thereby systematic comparison of their efficiency based on DNA yield, purity, 16S rRNA gene sequencing, and identification to determine the optimal DNA extraction methods whose DNA products reflect targeted bacterial communities special to rumen.

## Materials and Methods

### Ethics approval

Not applicable as the sample was brought from Government approved slaughterhouse.

### Sample collection and processing

Fresh rumen sample was brought from Government approved slaughterhouse near Pune, Maharashtra, India, under controlled environmental conditions. The sample was either used immediately for DNA extraction or stored at −80°C for 24-48 h without any preservatives. To evaluate the effect of long-term storage, the sample was stored at −80°C for 1.5 months and was studied further. For processing, rumen digesta was squeezed and washed with the artificial saliva, which consists of NaHCO_3_, 9.80 g/l; Na_2_HPO_4_, 4.97 g/l; KCl, 0.57 g/l; NaCl, 0.47 g/l; MgCl_2_, 0.123 g/l; and CaCl_2_, 0.04 g/l. Before use, saliva was heated at 39°C and infused with CO_2_ for anaerobicity. Squeezed and solid rumen digesta were processed separately for DNA extraction. Solid rumen digesta was centrifuged at 12,000 rpm/10 min, 4°C. The solid and liquid fractions were used separately. In the case of squeezed rumen digesta, the solid fraction was used after centrifugation. Both fractions were washed further with NaCl (HiMedia, India)-EDTA (Sigma-Aldrich) (1.5 M) thrice, before DNA extraction.

### Community DNA extraction

#### EM

EM1

1.5 g of fresh solid rumen digesta was centrifuged at 12,000 rpm/10 min, 4°C. Pellet and liquid fractions were separated before DNA extraction and washed with 1 ml 0.03 M NaCl-0.002M EDTA (pH: 8) followed by centrifugation at 12,000 rpm/5 min, 4°C. After three washing cycles, fractions were suspended in 100 µl NaCl-EDTA solution and 100 µl freshly prepared lysozyme (10 mg/ml, HiMedia, India). It was mixed thoroughly and incubated at 37°C for 1 h. It was followed by addition of 50 µl (10% SDS, Sigma-Aldrich), 10 µl proteinase K (Sigma-Aldrich), and 0.03 M NaCl-0.002M EDTA (pH: 8.0) to have total volume of 500 µl, followed by incubation at 55°C for 1 h.

After incubation, an equal amount of chloroform: isoamyl alcohol (24:1) was added to both fractions, mixed thoroughly and incubated at room temperature for 10 min. Whole content was centrifuged at 12,000 rpm/15 min, 4°C. The supernatant containing crude DNA was processed twice in a similar manner. 400 µl isopropanol (Sigma-Aldrich) and 7.5M ammonium acetate (HiMedia, India) were added into the washed liquid and incubated overnight at −20°C. Pellet was seen visually after the incubation. Entire content was centrifuged at 10,000 rpm/20 min, 4°C. Pellet obtained was washed thrice with 70% ethanol (Fisher Scientific) by centrifugation at 12,000 rpm/20 min, 4°C. Ethanol was removed completely after washings, and purified DNA was dried in a vacuum dryer (Thermo Fischer Scientific) without heating. Finally, the pellet was suspended in chilled 1× TE buffer (Invitrogen) and its quality was checked on a NanoDrop spectrophotometer (Thermo Fischer Scientific).

EM2

1.5 g of solid and 2 ml of squeezed rumen digesta were centrifuged at 12,000 rpm/10 min, 4°C. Their solid fractions were washed with 1 ml of 0.03M NaCl-0.002M EDTA (pH: 8) by centrifugation at 12,000 rpm/5 min, 4°C. They were suspended in 100µl 0.03M NaCl-0.002M EDTA (pH: 8) and a variable concentration of freshly prepared lysozyme (15 mg/ml, 20 mg/ml, and 25 mg/ml, HiMedia, India) followed by thorough mixing and incubation at 37°C for 1 h. Remaining steps were carried out as mentioned above for method EM1.

EM3

50 mg to 1000 mg of solid rumen digesta was centrifuged at 12,000 rpm/10 min, 4°C. Their solid fractions were washed with 1ml of 0.03M NaCl-0.002M EDTA (pH: 8) by centrifugation at 12,000 rpm/5 min, 4°C. After three washing cycles, they were suspended in 100 µl of 0.03M NaCl-0.002M EDTA (pH: 8) and 100 µl freshly prepared lysozyme (10 mg/ml, HiMedia, India). They were mixed thoroughly and incubated at 37°C for 1 h. Remaining steps were carried out as mentioned above for method EM1.

#### Chemical method (CM)

CM1

1.5 g solid rumen digesta was centrifuged at 12,000 rpm/10 min, 4°C. Pellet and liquid fractions were processed separately for DNA extraction. 2 ml liquid fraction was used in this study. They were washed with 1 ml 0.03M NaCl-0.002M EDTA (pH: 8) by centrifugation at 12,000 rpm/5 min, 4°C. After three washing cycles, fractions were suspended in lysis solution containing 100 mM Tris HCl (pH: 8.0, HiMedia, India), 20 mM EDTA (HiMedia, India), 1.4M NaCl (HiMedia, India), and 2% w/v CTAB (HiMedia, India) and incubated at 65°C for 1 h. Once brought to room temperature, whole content was centrifuged at 12,000 rpm/10 min, 4°C. The supernatant containing crude DNA was suspended in equal volume of fresh and chilled Chloroform: isoamyl alcohol (24:1) and incubated at room temperature for 10 min followed by centrifugation at 12,000 rpm/15 min, 4°C. After three washing cycles, remaining steps were carried out as mentioned in the method EM1.

CM2

1.5 g solid rumen digesta was processed as described in CM1. Their solid fraction was used for DNA extraction. It was added with 100 mM Tris HCl (pH: 8.0, HiMedia, India), 20 mM EDTA (HiMedia, India), 1.4M NaCl (HiMedia, India), and 0.2 to 1% w/v CTAB (HiMedia, India) lysis solution followed by incubation at 65°C for 1 h. After incubation, remaining steps were carried out as mentioned in the method EM1.

CM3

1.5 g solid rumen digesta was processed as described in CM1. Its solid fraction was used for DNA extraction. It was added with 2% w/v CTAB (HiMedia, India) followed by incubation at 65°C for 30 min. After incubation, remaining steps were carried out as mentioned in the method EM1.

CM4

1.5 g solid rumen digesta was processed as described in CM1. Its solid fraction was used for DNA extraction. It was added with 2% w/v CTAB (HiMedia, India) followed by incubation at 65°C for 2 h. After incubation, remaining steps were carried out as mentioned in the method EM1.

#### Enzymatic+Chemical method (ECM)

ECM1

1.5 g solid and 2 ml of squeezed rumen digesta were processed as described in method EM1. Their solid fractions were used for DNA extraction. Fractions lysed with enzymatic method were treated with lysis solution containing 100 mM Tris HCl (pH: 8.0, HiMedia, India), 20 mM EDTA (HiMedia, India), 1.4M NaCl (HiMedia, India), and 2% w/v CTAB (HiMedia, India). It was incubated at 65°C for 10 min. Remaining steps were carried out as mentioned in the method EM1.

#### Enzymatic+Chemical+Physical method (ECPM)

ECPM1

1.5 g solid and 2 ml of squeezed rumen digesta were processed as described in method EM1. Their solid fractions were used for DNA extraction followed by enzymatic lysis using lysozyme as described earlier. They were added with lysis solution containing 100 mM Tris HCl (pH: 8.0, HiMedia, India), 20 mM EDTA (HiMedia, India), 1.4M NaCl (HiMedia, India), and 2% w/v CTAB (HiMedia, India). It was mixed with 50 µl (10% SDS, Sigma-Aldrich) followed by quick freezing of the content at −80°C for 10 min and thawing at 65°C. After three freeze-thaw cycles, content was brought at room temperature. It was centrifuged at 12,000 rpm/10 min, 4°C. The supernatant containing crude DNA was suspended in equal volume of fresh and chilled Chloroform: isoamyl alcohol (24:1) and incubated at room temperature for 10 min followed by centrifugation at 12,000 rpm/15 min, 4°C. After three washing cycles, remaining steps were carried out as mentioned in the method EM1.

ECPM2

This was performed similarly as ECPM1 as described above. ECPM2 was performed differently where 2.0 % w/v PVP (PVP 40, Sigma-Aldrich) was added to the lysis solution, before three freeze-thaw cycles. Remaining steps were carried out as mentioned in the method ECPM1.

ECPM3

2 ml of squeezed rumen digesta was used for DNA extraction. This was performed similarly as ECPM1 as described above. ECPM3 was different where lysis solution was supplemented with 2.0 % w/v PVP (PVP 40, Sigma-Aldrich), and the number of freeze-thaw cycles was increased from three to eight. Remaining steps were carried out as mentioned in the method ECPM1.

### Community DNA extraction with QIAamp DNA Stool Mini Kit (Qiagen, 51504)

200 mg of solid and 200 µl of squeezed rumen digesta were used for DNA extraction using QIAamp DNA Stool Mini Kit (Qiagen, 51504). Effect of lysis temperature (70°C and 90°C for 5 min) on community DNA extraction was also evaluated. Remaining steps were performed according to the instructions provided by the manufacturer.

### Evaluation of reliability and reproducibility of CM4 and ECPM2 methods for community DNA extraction

1.5 g of solid and 2 ml squeezed rumen digesta were used for statistical analysis of CM4 and ECPM2 methods, respectively. Details of the methods were as described above. Three independent trials were conducted with fresh solid and squeezed rumen digesta for assessing its reproducibility. Each trial was conducted in eight different vials for analyzing its reliability. Furthermore, an independent trial was conducted with −80°C stored, 1 day old, and 1.5-month-old rumen digesta using CM4 and ECPM2 methods. Statistical analysis was performed using mean and standard deviation.

### Yield, purity, and integrity of DNA

DNA concentrations were measured on NanoDrop spectrophotometer (Thermo Fisher Scientific). The purity of DNA was assessed spectrophotometrically from A_260nm_/A_280nm_ to A_260nm_/A_230nm_ ratios to indicate the presence of buffer salts and organic compounds as a part of DNA contamination. Integrity was determined by agarose (2% w/v) gel electrophoresis (2 h, 10 cm×15 cm Mini-Sub^®^ Cell GT, Bio-Rad) at 140 V using 1 Kb Plus DNA Ladder (N3232S, NEB) as a molecular weight marker, post-staining with SYBR^®^ Safe DNA gel stain (Invitrogen) and illumination under UV light.

### Assessing the suitability of Community DNA for PCR-based rumen microbial ecology applications

#### Standard PCR using 16S rRNA universal bacterial primers

The purity of community DNA for downstream applications was assessed by amplification of 16S rRNA using the universal bacterial primers as (27F 5’ AGAGTTTGATCMTGGCTCAG 3’ and 1492R 5’ TACGGYTACCTTGTTACGACTT 3’). PCR amplification was done using a 20 µl reaction mixture containing 0.2 mM each dNTP (N0446S, New England Biolabs), 0.5 µM of each forward and reverse primer, 100 ng DNA template, and 0.02 U/µl of Q5 Hot Start High Fidelity DNA polymerase (New England Biolabs) with 1× reaction buffer supplied by the manufacturer. Amplification was performed with a thermal cycler (Bio-Rad) using the following program: 98°C for 10 min; 35 cycles consisting of 98°C for 10 s; 55°C for 30 s; 72°C for 1 min; and a final extension step consisting of 72°C for 10 min. The amplification was determined by electrophoresis of reaction product in 1% agarose gel.

#### Standard PCR using 16S rRNA primers for targeted rumen bacteria

Community DNA samples showing PCR amplification with 1.5 Kb band were used for standard PCR for assessing the presence of targeted rumen bacteria using specific bacterial 16S rRNA primers, Q5^®^ Hot Start High-Fidelity DNA Polymerase (M0493, New England Biolabs), 0.2 mM of each dNTPs (N0446S, New England Biolabs), 100 ng template, and 10 nM of forward and reverse primer (Table-1) [10,11]. PCR was performed in 20 µl reaction volume using the following program: 98°C for 10 min; 35 cycles consisting of 98°C for 10 s; optimized annealing temperature as given in (Table-1) for 30 s; 72°C for 1 min; and a final extension step consisting of 72°C for 10 min. The amplification was determined by electrophoresis of reaction product in 2% agarose gel.

**Table-1:** PCR primers used to detect targeted rumen bacteria.

Primer	Sequence (5’- 3’)	Annealing temperature T_a_ (°C)*	Target	Amplicon size (bp)	References
Bac 303f	GAAGGTCCCCCACATTG	61	Genus *Bacteroides* and *Prevotella*	418	Stiverson *et al.* [10]
Bac 708r	CAATCGGAGTTCTTCGTG				
Fs-f	GGTATGGGATGAGCTTGC	64	*F. succinogenes*	446	Stiverson *et al.* [10]
Fs-r	GCCTGCCCCTGAACTATC				
Sel-Mit-f	TGCTAATACCGAATGTTG	57	*S. ruminantium* and *M. multacida*	513	Stiverson *et al.* [10]
Sel-Mit-r	TCCTGCACTCAAGAAAGA				
SB-JCM5802-f	CTAATACCGCATAACAGCAT	58	*S. bovis*	869	Tajima *et al*. [11]
SB-JCM5802-r	AGAAACTTCCTATCTCTAGG				
RF-ATCC19208-f	GGACGATAATGACGGTACTT	62	*R. flavofaciens*	835	Tajima *et al.* [11]
RF-ATCC19208-r	GCAATCCGAACTGGGACAAT				

* T_a_ mentioned in above table is calculated as per NEB resources and tools. PCR=Polymerase chain reaction, *F. succinogenes=Fibrobacter succinogenes*, *S. ruminantium=Selenomonas ruminantium*, *M. multacida=Mitsuokella multacida*, *S. bovis=Streptococcus bovis*, *R. flavofaciens=Ruminococcus flavefaciens*

Methods which performed consistently to give intact, good quality DNA with clean PCR bands for all targeted bacteria were finalized further for 16S rRNA gene sequencing and identification studies.

### 16S rRNA gene sequencing and identification

PCR product of each sample was subjected to electrophoresis on 2% agarose gel. 418 bp, 862 bp, 835 bp, 513 bp, and 446 bp fragments were observed and eluted from the gel using Macherey Nagel NucleoSpin^®^ Gel and PCR Clean-up Kit (740609.50 MN). The forward and reverse primers were used in sequencing of the eluted PCR products; the sequencing process was done in First Base Malaysia by Sanger sequencing technique. The obtained sequences were analyzed by a BLASTN search in NCBI GenBank. A phylogenetic tree was constructed using sequence distance method.

## Results

**Figure-1:**
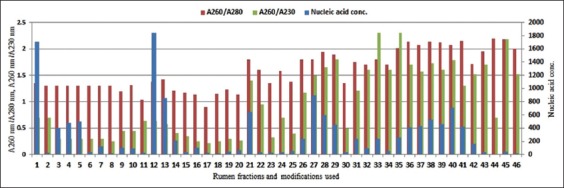
Efficacy of modified community DNA extraction methods. Nucleic acid concentration, A_260nm_/A_280nm_ and A_260nm_/A_230nm_ ratios obtained using various rumen fractions and methods modifications including, 1-2: Enzymatic method (EM)1 solid-S and EM1-solid-L (10 mg/ml lysozyme), 3-5: EM2 solid (15 mg/ml, 20 mg/ml and 25 mg/ml lysozyme), 6-8: EM2 squeezed (15 mg/ml, 20 mg/ml and 25 mg/ml lysozyme), 9-20: EM3-solid (50 mg, 100 mg, 150 mg, 200 mg, 350 mg, 400 mg, 500 mg, 600 mg, 700 mg, 800 mg, 900 mg, and 1000 mg sample size), 21-22: Chemical method (CM)1-solid-S and CM1-Solid-L (1% CTAB 65°C/1 h), 23-27: CM2-solid (0.2-1% CTAB with 65°C/1 h), 28: CM3-solid (1% CTAB, 65°C/30 min), 29: CM4-solid (1% CTAB, 65°C/2 h), 30-31: Enzymatic+Chemical method (ECM)1-solid and ECM1-squeezed, 32-33: Enzymatic+Chemical+Physical method (ECPM)1-solid and ECPM1-squeezed, 34-35: ECPM2-solid and ECPM2-squeezed (with 0.8 g PVP), 36-42: ECPM3-squeezed (with 0.8 g PVP and 3-8 freeze-thaw cycles), 43-44: QI-solid and QI-squeezed (with 70°C lysis), and 45-46: QI-solid and QI-squeezed (with 90°C lysis).

**Figure-2:**
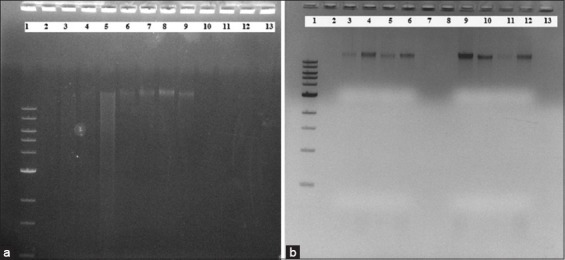
(a) Community DNA extraction with Enzymatic method (EM)1, Enzymatic-Chemical method (ECM)1 and Enzymatic+Chemical+Physical method (ECPM2) methods. Community DNA extraction with >15 Kb band on 1% agarose gel electrophoresis using (b) ECPM2, EM1, and ECM1 method, Lane 1: Ladder: 1 Kb, New England Biolabs. Community DNA extraction with ECPM2 using Lane 2-5: Solid rumen digesta, Lane 6-9: Squeezed rumen digesta, EM1 using Lane 10: Solid rumen digesta, Lane 11: Blank, ECM1 using Lane 12: Solid rumen digesta, and Lane 13: Squeezed rumen digesta. (b) Community DNA extraction with CM4 method. Community DNA extraction with >15 Kb band on 1% agarose gel electrophoresis using (a) CM4 method, Lane 1: Ladder: 1 Kb, New England Biolabs, Lane 2: Blank, Lane 3-6: Community DNA with CM4 method, Lane 7-8: Blank, Lane 9-12: Community DNA with CM4 method, and Lane 13: Blank.

This work was aimed at the development of PCR and 16S rRNA gene sequencing quality community DNA extraction protocols through modification in existing methods followed by their optimization. In this instance, details of four different methods of community DNA extraction modified in various ways are shown in Table-2a to e and compiled together in Figure-1. Modified Enzymatic methods studied in three different sets of EM1, EM2, and EM3 differed in their nucleic acid yield (ng/µl) through their A_260nm_/A_280nm_ and A_260nm_/A_230nm_ ratios were similar in the range of 1.3 and 0.5, respectively. EM1 processed with 10 mg/ml lysozyme, and solid-liquid fractions of rumen digesta could extract 1710 ng/µl and 30 ng/µl nucleic acid. Whereas solid and squeezed rumen digesta processed with 15 mg/ml, 20 mg/ml, and 25 mg/ml lysozyme in EM2 could extract successively higher nucleic acid with increasing concentration of lysozyme, where 25 mg/ml lysozyme could give 500 ng/µl and 180 ng/µl nucleic acid from solid and squeezed rumen digesta. In EM3, varying sample size from 50 mg to 1000 mg could extract highest nucleic acid of 1844 ng/µl from 200 mg. However, purity ratios of A_260nm_/A_280nm_ and A_260nm_/A_230nm_ obtained were poor for any set of modifications conducted in enzymatic method. Suspension of extracted community DNA was brownish, non-uniform. Furthermore, it failed to show community DNA on agarose gel electrophoresis Figure-2a).

**Table-2a:** Yield and purity of community DNA extracted using modified Enzymatic method.

Modified Methods studied	Rumen fraction used	Quantity of rumen fraction used	Lysozyme conc. (mg/ml)	Nucleic acid conc. (ng/µl)*	A_260nm_/A_280nm*_	A_260nm_/A_230nm*_
EM1	Solid	1.5 g	10	1710	1.35	0.7
	Liquid	2 ml	10	30	1.3	0.7
EM2	Solid	1.5 g	15	400	1.3	0.3
		1.5 g	20	480	1.3	0.3
		1.5 g	25	500	1.3	0.3
	Squeezed	2 ml	15	40	1.3	0.3
		2 ml	20	120	1.3	0.3
		2 ml	25	180	1.3	0.25
EM3	Solid	50 mg	10	101	1.19	0.44
		100 mg	10	92	1.32	0.44
		150 mg	10	25	1.04	0.64
		200 mg	10	1844	1.38	0.64
		350 mg	10	859	1.42	0.58
		400 mg	10	213	1.21	0.41
		500 mg	10	31.4	1.17	0.35
		600 mg	10	100	1.13	0.25
		700 mg	10	31	0.9	0.22
		800 mg	10	20	1.15	0.25
		900 mg	10	42	1.23	0.3
		1000 mg	10	62	1.13	0.26

* Data value represents mean of duplicates for each method. EM=Enzymatic method

Modified chemical method studied in four different sets of CM1, CM2, CM3, and CM4 not only showed varying yield of nucleic acid (ng/µl) but also A_260nm_/A_280nm_ and A_260nm_/A_230nm_ ratios. CM1 processed with solid and liquid fractions of rumen digesta with 2% CTAB could extract 644 ng/µl and 40 ng/µl nucleic acid. Community DNA extraction with solid fraction and increasing concentration of 0.2-1%, CTAB was performed with CM2 where; increasing concentration of CTAB could extract successively higher nucleic acid with better purity ratios and 1% CTAB was found to be the most promising concentration. Nonetheless, CM3 and CM4 with 2% CTAB application followed by 30 min and 2 h lysis buffer incubations resulted into nearest optimal ratios of A_260nm_/A_280nm_ and A_260nm_/A_230nm_ for the extracted community DNA. In the application of CM4, DNA suspension was white, translucent and it showed an intact band of >15 Kb on 1% agarose gel electrophoresis (Figure-2b).

ECM studied with solid and squeezed rumen digesta could extract 40 ng/µl and 240 ng/µl nucleic acid; indicating about better efficacy of EC method toward squeezed rumen digesta. However, the purity ratios of A_260nm_/A_280nm_ and A_260nm_/A_230nm_ shown below optimum and it also failed to show community DNA on agarose gel electrophoresis (Figure-2a). Suspension of community DNA obtained was brownish, non-uniform. Overall, EC method could not extract a good quality community DNA.

ECPM of community DNA extraction comprises three different modifications where ECPM1 and ECPM2 were processed with solid and squeezed rumen digesta, distinctly with 2% PVP 40 application for ECPM2. Both methods could extract 240 versus 260 ng/µl and 90 versus 60 ng/µl community DNA from solid and squeezed rumen digesta. Increasing number of freeze-thaw cycles from three to eight could not increase the yield of community DNA as seen in ECPM3. A_260nm_/A_230nm_ ratio seen inclined toward non-optima with increasing freeze-thaw cycles though A_260nm_/A_280nm_ seen consistent in the range of 2–2.1. Overall, squeezed rumen digesta seen very suitable for ECP method of community DNA extraction for any set of modifications conducted as described in ECPM1, ECPM2, and ECPM3 (Figure 2a). Ratio of A_260nm_/A_280nm_ of 1.7 for solid rumen digesta indicates its complexity and non-feasibility toward ECPM based community DNA extraction protocols.

Community DNA extraction using QIAamp DNA Stool Mini Kit (Qiagen, 51504) followed by 70°C and 90°C lysis incubation could give highly efficient cell lysis with optimal purity ratios at 90°C. Irrespective of lysis temperature used, solid fraction could extract higher nucleic acid than squeezed rumen digesta which was 40 ng/µl and 10 ng/µl, respectively (Table 2e).

**Table-2b:** Yield and purity of community DNA extracted using modified Chemical method.

Modified methods studied	Rumen fraction used	CTAB conc. (% w/v)	Time (h)	Nucleic acid conc. (ng/µl)*	A_260nm_/A_280nm*_	A_260nm_/A_230nm*_
CM1	Solid	2	1	644	1.8	1.4
	Liquid	2	1	40	1.6	0.95
CM2	Solid	0.2	1	30	1.35	0.32
		0.4	1	40	1.58	0.7
		0.6	1	60	1.38	0.4
		0.8	1	240	1.8	1.17
		1	1	900	1.8	1.5
CM3	Solid	2	30 min	600	1.94	1.65
CM4	Solid	2	2	450	1.9	1.8

* Data value represents mean of duplicates for each method. CTAB=Cetrimonium bromide, CM=Chemical Method

**Table-2c:** Yield and purity of community DNA extracted using modified ECM.

Modified methods studied	Rumen fraction used	Nucleic acid conc. (ng/µl)*	A_260nm_/A_280nm*_	A_260nm_/A_230nm*_
ECM1	Solid	40	1.35	0.49
	Squeezed	240	1.75	1.2

* Data value represents mean of duplicates for each method. ECM=Enzymatic+Chemical method

**Table-2d:** Yield and purity of community DNA extracted using modified ECPM.

Modified methods studied	Rumen fraction used	Number of freeze-thaw cycles	Nucleic acid conc. (ng/µl)*	A_260nm_/A_280nm*_	A_260nm_/A_230nm*_
ECPM1	Solid	3	90	1.7	1.6
	Squeezed	3	240	1.8	2.3
ECPM2	Solid	3	60	1.7	1.6
	Squeezed	3	260	1.8	2.3
ECPM3	Squeezed	3	435	2.14	1.7
		4	433	2.08	1.7
		5	450	2.13	1.73
		6	460	2.12	1.6
		7	465	2.07	1.58
		8	424	2.15	1.3

* Data value represents mean of duplicates for each method. ECPM=Enzymatic+Chemical+Physical method

**Table-2e:** Yield and purity of community DNA extracted using modified QIAamp DNA Stool Mini Kit (Qiagen, 51504).

Kit method used	Rumen fraction used	Quantity of rumen fraction used	Lysis temperature studied	Nucleic acid conc. (ng/µl)*	A_260nm_/A_280nm*_	A_260nm_/A_230nm*_
QIAamp DNA Stool Mini Kit (Qiagen, 51504)	Solid	200 mg	70°C	40.1	1.95	1.7
	Squeezed	200 µl	70°C	10.1	2.2	0.7
	Solid	200 mg	90°C	47.3	2.19	2.18
	Squeezed	200 µl	90°C	13.2	2	1.52

* Data values represent mean of duplicates for each method

Irrespective of CM4 or ECPM2 methods application, statistical analysis (Table-3) showed a drastic drop in purity level of DNA by increasing sample storage period from day 0 to 1.5 months. Here, A_260nm_/A_280nm_ and A_260nm_/A_230nm_ ratio of day 0 was observed to be 1.8± 0.06 and 2.3±0.83 for CM4 method. It was seen to be 1.8±0.075 and 2.3±0.18 for ECPM2 method. Whereas, A_260nm_/A_280nm_ and A_260nm_/A_230nm_ of 1.5 month stored rumen digesta seen reduced from 1.8± 0.06 to 1.72±0.15 and 1.43±0.23, 2.3±0.83 to 1.27±0.41 and 0.62±0.17 for CM4 and ECPM2 methods respectively. In common, nucleic acid concentration showed the highest mean standard deviation values irrespective of the storage period of solid rumen digesta.

**Table-3:** Statistical analysis of optimized CM4 and ECPM2 methods.

Community DNA extraction method	Analysis parameter	Storage period of solid rumen digesta (at−80°C)					

		Fresh		1 day		1.5 months	
		
		Mean	Mean SD	Mean	Mean SD	Mean	Mean SD
CM4	Nucleic acid (ng/µl)	835	714	459	417	811	436
	A_260nm_/A_280nm_	1.8	0.06	2.08	0.32	1.72	0.15
	A_260nm_/A_230nm_	2.3	0.83	2.03	0.63	1.27	0.41
ECPM2	Nucleic acid (ng//µl)	161	108	112	54	456	135
	A_260nm_/A_280nm_	1.8	0.075	1.86	0.086	1.43	0.23
	A_260nm_/A_230nm_	2.3	0.18	2.02	0.15	0.62	0.17

**Figure-3:**
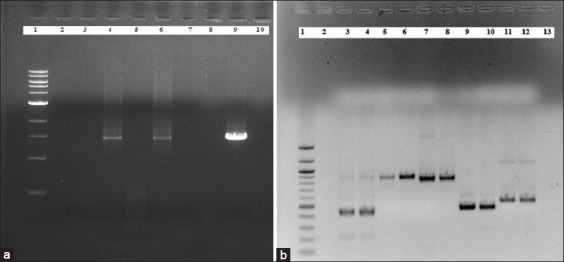
(a and b) Standard polymerase chain reaction (PCR) using community DNA extracted using modified methods. Standard PCR using (a) universal bacterial primers with community DNA extracted using Lane 1: 1 Kb ladder (New England Biolabs), Lane 2: Enzymatic method (EM)1, Lane 3: EC1, Lane 4: Enzymatic+Chemical+Physical method (ECPM)2, Lane 5: Chemical method (CM)1, Lane 6: CM4, Lane 7: CM2 methods, Lane 9: Genomic DNA of *Bacillus subtilis* as a positive control and Lane 10: blank (b) Specific targeted bacterial 16S rRNA gene primers and community DNA as follows. Lane 1: 100 bp ladder (New England Biolabs), Lane 2: Blank, Lane 3-4: CM4 and ECPM2 DNA with genus *Bacteroides* and *Prevotella* (418 bp), Lane 5-6: CM4 and ECPM2 DNA with *Streptococcus bovis* (869 bp), Lane 7-8: CM4 and ECPM2 DNA with *Ruminococcus flavefaciens* (835 bp), Lane 9-10: CM4 and ECPM2 DNA with *Fibrobacter succinogenes* (446 bp), and Lane 11-12: CM4 and ECPM2 DNA with *Selenomonas ruminantium* (513 bp), and Lane 13: blank.

Community DNA belongs to ECPM2 and CM4 method given 1.5 Kb band on 1% agarose gel electrophoresis (Figure-3a) in standard PCR using 16S rRNA universal bacterial primers. More interestingly, community DNA extracted with both methods have given PCR amplification for genus *Bacteroides* and *Prevotella* (418 bp), *Streptococcus bovis* (869 bp), *Ruminococcus flavofaciens* (835 bp), *Fibrobacter succinogenes* (446 bp), and *Selenomonas ruminantium* (513 bp) with targeted bacterial 16S rRNA gene primers (Figure-3b) and optimized annealing temperature (Table-1). Moreover, 16S rRNA gene sequencing and identification confirmed the presence of *P. rumnicola, S. lutetiensis, R. flavofaciens, F. succinogens*, and *S. ruminantium* in goat rumen. However, genus *Bacteroides* and *Mitsuokella multacida* were not detected in this study. NCBI BLAST analysis revealed 95%, 99%, 98%, 99%, and 97% sequence similarity with targeted rumen bacteria such as *P. ruminicola, S. ruminantium, R. flavofaciens, S. lutetiensis*, and *F. succinogenes*, respectively.

## Discussion

The extreme complexity of rumen microbiota is being understood and solved through various approaches of molecular biology and metagenomics for which good quality community DNA is of prime importance. Although methods can vary, all ascertain three major aims including comprehensive cell lysis, removal of non-nucleic acid components, and minimal loss of extracted nucleic acid [12]. In context to this, bead beating mediated cell lysis, and phenol-based DNA purification is among established protocols of many researchers [5,13]. Jin *et al*. [14] and Stiverson *et al*. [10] applied bead beating method and RBBC method for community DNA extraction. Whereas, Minas *et al*. [15], Popova *et al*. [16], and Bashir *et al*. [17] used phenol for community DNA purification. The present study does not recommend using bead beating and phenol. Rumen digesta of slaughtered goat was used to evaluate an effect of modified methods on its different fraction. Finally, the fractions giving optimal results were used for method optimization.

In this instance, EM1, EM2, and EM3 methods could not extract optimal quality community DNA from any of the fraction such as solid, liquid, or squeezed rumen digesta; though 10 mg/ml lysozyme application resulted into the highest yield of nucleic acid (ng/µl) from solid fraction. This emphasizes on the strongest association of microbial community with solid rumen digesta, where they must be present in highest abundance. Increasing lysozyme concentration up to 25 mg/ml could not improve it further which marks its poor efficacy in conclusion. Furthermore, the increase in sample size showed decreased yield which is a sign of increasing concentration of inhibitors. Here, 200 mg sample size found most suitable.

In continuation to this; brownish, non-uniform DNA suspension followed by smearing in the agarose gel electrophoresis is indicative of non-feasibility of an enzymatic method for community DNA extraction. Rumen content samples contain many substances, such as tannins, that could inhibit the PCR. Relative absorbance readings (A_260nm_/A_230nm_ for carbohydrates, aromatic compounds, humic acids, and phenolics; A_260nm_/A_280nm_ for protein) provide an indication of DNA purity and should ideally be 2.0 to 2.2 for A_260nm/_A_230nm_ and 1.8 for A_260nm_/A_280nm_ for most molecular biology applications [2,18]. Chen *et al*. [19] could get A_260nm_/A_280nm_ of 1.8 with community DNA extraction from Yak rumen digesta using SDS-lysozyme method. A_260nm_/A_280nm_ obtained in our study could not exceed beyond 1.3. This might indicate variable efficacy of community DNA extraction method in each different ruminant and respective rumen fractions.

To accomplish optimal quality DNA extraction, the chemical method performed in four sets of modifications such as CM1, CM2, CM3, and CM4 could extract community DNA with optimal purity ratios very efficiently. These methods are different from Henderson *et al*. [2], Yu and Morrison [20] which preferred column based DNA purifications. In continuation with this, Popova *et al*. [16] and Fliegerova *et al*. [21] applied bead beating in merge with community DNA technique employed. In our study, lysis buffer incubation with solid fraction at 65°C for 30 min, 1 h and 2 h without bead beating application successively increased the nucleic acid concentration along with its purity. Furthermore, 0.2%-1% CTAB variation could give similar results where 1% CTAB found most efficient. 1% CTAB seems most suitable for removal of strong inhibitors which can otherwise affect the DNA integrity. Unlike to the enzymatic method, the chemical method could extract uniform, white and translucent community DNA which showed an intact band on agarose gel electrophoresis. 2% CTAB with 2 h incubation seems highly effective for removal of tannin and polyphenols which can affect the DNA quality and integrity. Comparative analysis of enzymatic and chemical method draws the major conclusion toward association of large abundance of microbial communities with a solid fraction of goat rumen digesta, followed by squeezed and liquid fractions.

In accordance with this, a combination of enzymatic and chemical method could not fulfill the objective of extracting community DNA with highest efficiency and performance. In contrast to enzymatic and chemical methods, their combination together could extract highest nucleic acid from squeezed rumen digesta where solid fraction could give average yield. However, similar to enzymatic method, it could extract community DNA having brownish, non-uniform suspension which failed to give a band on agarose gel electrophoresis. It seems that independent enzymatic method or its combination with chemical method may not give functional community DNA as required for molecular biology applications.

Combination of Enzymatic+ Chemical + Physical method drawn a similar conclusion to EC method wherein highest nucleic acid was obtained from squeezed rumen digesta but with optimal purity ratios. Increase in a number of freeze-thaw cycles as a part of its modification could not improve it to a large extent. Three freeze-thaw cycles and 2% w/v PVP 40 applications for squeezed rumen digesta achieved the optimal performance. Along with chemical method, it proved to be the second effective method to give community DNA extraction efficiently. Furthermore, suitability of ECPM2 toward squeezed rumen digesta may suggest its efficacy for distinct microbial consortia other than solid fraction. Collectively, CM4 and ECPM2 methods can possibly extract a good quality community DNA even without application of RNAase, phenol or any kit based column purifications. QIAamp DNA Stool Mini Kit (Qiagen, 51504) the only kit method studied here; could give highest nucleic acid in solid fraction than squeezed rumen digesta. Furthermore, lysis temperature of 90°C found most effective for efficient cell lysis. High temperature may help in efficient cell wall degradation of Gram-positive bacterial communities and thereby improve the yield.

Thus, community DNA extraction performed using QIAamp DNA Stool Mini Kit (Qiagen, 51504), methods EM1 and CM4 found most effective with solid rumen digesta. Methods ECM1 and ECPM2 showed efficient performance with squeezed rumen digesta. Apart from this, 1.5 months stored rumen sample showed a statistically significant drop in purity ratios of community DNA. The choice of DNA extraction method followed by sampling and storage may have an impact on the revealed community structure [22]. This is in agreement with Tatiana *et al*. [9] which shown that rumen sample storage can have influence over the yield of metagenomics DNA, abundances of specific phyla, class or other taxa and thus can change the picture of diversity indices and community richness. Although the change in diversity richness has been recorded for rumen samples, it’s may not be the case for soil community DNA extraction where no any significant differences have been noticed in samples processed after preservation with respect to their DGGE profiles or DNA quality [23]. This emphasizes on criticality of rumen samples and methods to follow in.

Following this, community DNA extracted with CM4 and ECPM2 methods could give standard PCR amplification with universal bacterial primers and targeted bacterial 16S rRNA gene primers which prove its efficacy for a variety of molecular biology applications. Furthermore, PCR amplification of targeted bacteria with solid fraction and squeezed fraction of goat rumen digesta shows their strong association with rumen. It may denote different compatibility of each of the fraction for specific extraction method employed. 16S rRNA gene sequencing and identification of PCR amplified products confirmed the presence of *R. flavofaciens*, *F. succinogenes*, *S. ruminantium*, and *S. lutetiensis* in goat rumen. Along with this, *P. ruminicola* was detected against targeted genus *Bacteroides* and *Prevotella*. PCR and 16S rRNA based culture independent analyses are thus very informative and necessary to understand population wide community patterns [24-27]. In contrast, *M. multacida* was not detected in this study. This is in agreement with Pers-Kamczyc *et al*. [28] and Li *et al*. [29] which stated that the presence of rumen microorganisms may vary as per their associated functions. This leaves a possibility of the presence of these undetected genera and species in different rumen fraction or current optimized methods may require extreme sensitive operations for their detection. In another way, noticing their presence through molecular biology needs more attention and methods establishment.

## Conclusion

To the best of our knowledge, this is the first report of optimization of modified CTAB and CTAB-SDS-PVP-lysozyme-freeze-thaw methods for community DNA extraction from solid rumen digesta, its centrifuged supernatant and squeezed rumen digesta of a slaughtered goat. Furthermore, this study showed that short-term rumen sample preservation at −80°C without any external preservatives does not affect the quality of community DNA though long-term sample preservation for 1.5 months does. Methods optimized in our study do not recommend the use of phenol, RNAase, proteinase K or kit based DNA purifications which differentiate them from others. Possible extraction of >15 Kb community DNA from rumen confirms their efficacy for molecular biology applications. Consistent purity ratios, high nucleic acid yield, and PCR amenability stand out the reliability and reproducibility of these methods. Further, detection of *P. ruminicola, S. lutetiensis, R. flavofaciens, S. ruminantium*, and *F. succinogenes* in solid and squeezed rumen digesta proves their strongest association with rumen fiber mat. It also marks the presence of distinct microbial communities in solid and squeezed rumen fractions that, in turn, differs the performance of each different method employed and yield of nucleic acid obtained. It also leaves a possibility of the presence of difficult microbial consortia in squeezed rumen digesta whose DNA extraction methods need more attention. Finally, manual protocols of community DNA extraction may vary in different ruminant which suggests undertaking rigorous research in the development of new protocols.

## Authors’ Contributions

DA and AK designed the research; DA collected the samples, performed the research, analyzed data and prepared the manuscript, AK guided the entire research, data analysis, and manuscript preparation. All authors read and approved the final manuscript.
